# Characteristics of Double Planar Micro-Inductor with Patterned NiFe Thin-Films for DC/DC Integration

**DOI:** 10.3390/mi8050151

**Published:** 2017-05-08

**Authors:** Mingming Chen, Guifu Ding, Ping Cheng, Congchun Zhang, Xiaomin Zhu, Zhe Liu

**Affiliations:** National Key Laboratory of Science and Technology on Micro/Nano Fabrication, Department of Micro/Nano Electronics, School of Electronic Information and Electrical Engineering, Shanghai Jiao Tong University, Shanghai 200240, China; cmm0319@sjtu.edu.cn (M.C.); small.sunshine.smile@sjtu.edu.cn (X.Z.); df2009lz@163.com (Z.L.)

**Keywords:** double planar, film inductor, micromachining, patterned magnetic film

## Abstract

This paper proposes a double-planar-coil microinductor with patterned permalloy magnetic film for high frequency DC–DC integration. The effects of magnetic film’s patterning and thickness on the inductance and quality factor of the micro-inductor are investigated by using COMSOL Multiphysics software. Simulation results indicate that the magnetic film improves the inductance of microinductor effectively and patterning of the magnetic film reduces eddy current loss in high frequency range. The micro-inductor is fabricated by using micro-electro-mechanical systems (MEMS) technique. The inductance of approximately 2.17 μH at 1.5 MHz and the quality factor of 2.8 are achieved for the microinductor with patterned magnetic film. The performances of the micro-inductor applied in a low-power DC/DC converter are tested. The results indicate that the micro-inductor with the patterned magnetic film effectively has improved inductance and quality factor compared to that with non-patterned magnetic film. The maximum efficiency of measured converter is 67% at 1.5 MHz and the output current is 100 mA.

## 1. Introduction

With the decrease of size and weight of portable electronic products, the requirement of a power management chip for internal integration is becoming urgent. For micro-processors and mobile devices, thin film magnetic inductors are useful for integrated power delivery solutions, which will replace conventional wire-wound inductors [1,2]. The miniaturization and integration of the inductor with electronic circuits are the key to realize electronic products with high performance, small size, light weight, high saturation current, high efficiency, and low lost [3,4,5]. In recent years, with the rapid development of micro-electro-mechanical systems (MEMS) fabrication process and integrated passive devices (IPD) technology, the thin-film technology combined with MEMS process becomes one of the most advanced technologies to develop micro-inductive devices [6,7,8]. Since Soohoo et al. proposed and prepared the micro-inductors with magnetic thin-film in 1979, many researchers have paid much attention to this field [9,10,11]. Sugawara et al. developed a monolithic DC/DC converter by utilizing a thin-film planar inductor and CoHfTaPd amorphous magnetic film. The inductance and quality factor of the inductor were 0.96 µH and *Q* = 4.3 [12].

The use of thin-film magnetic inductors has the advantages of increasing inductance per unit and decreasing containment of magnetic fields compared to coreless inductors. However, it has the disadvantage of extra eddy current loss in the magnetic film, especially when the inductor works in high frequency.

In general, the micro-inductors must have small size which is comparable to the size of the active components if they are integrated in DC/DC converters successfully. In order to reduce the eddy current losses resulted from the magnetic film in high frequency, a planar inductor with patterned magnetic film has been developed in this study. The patterned magnetic film can concentrate the magnetic field lines to improve inductance, which reduces eddy current losses to get high quality factor. The magnetic film was prepared by electrodepositing Ni_80_Fe_20_ permalloy which has relatively high saturation flux density (>0.6 T) and initial permeability *μ_i_* (40–60 mH/m). For comparison, an inductor without magnetic film and an inductor with non-patterned magnetic film were also fabricated. The three inductors have the same planar double coils. The performances at 1.5 MHz low-power DC/DC converter of the fabricated micro-inductors are presented. The design and simulation of the micro-inductor device are described in Section 2. Inductor fabrication and performance characteristics are described in Section 3. Section 4 highlights measurement results for the micro-inductor when used with a prototype 1.5 MHz DC/DC down-converter chip. Conclusions and future developments are presented in Section 5. 

## 2. Model Design and Simulation

**Figure 1:**
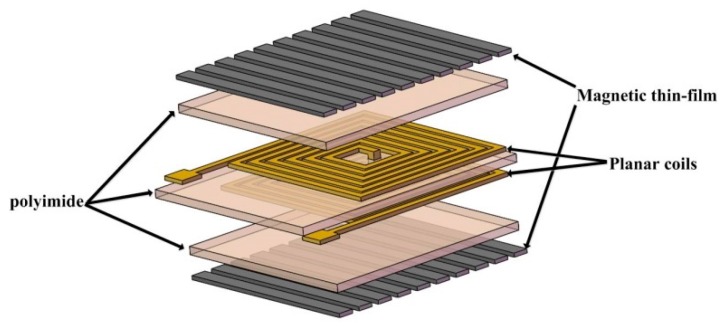
Structure of planar inductor with patterned magnetic film.

The inductance value can be calculated according to following formula [13].

(1) $$L=K_{1}\mu_{0}\frac{n^{2}d_{avg}}{1+K_{2}\rho }$$

where *L* is inductance of the inductor, with the unit of nH; n represents the turns of coil; *μ*_0_ is the magnetic permeability; *d_avg_* is the average diameter of the inductance coil; both *K*_1_ and *K*_2_ are constants, which are determined by the shape of the inductance coil; *ρ* represents the filling rate. From the Equation (1), it can be seen that increasing the coil’s turns can achieve large inductance. Besides, the inductance is also affected by the coil’s size. In order to achieve more turns in a small area, double planar spiral coils are adopted in the design. Figure 1 schematically shows the structure of planar micro-inductor with patterned magnetic film. The patterned magnetic film is expected to reduce eddy current loss at high frequency and to increase the quality factor of the micro-inductor. The slots in the magnetic film should be sufficiently narrow so that the vertical magnetic field cannot leak through the patterned magnetic film. The polyimide is selected as the dielectric. A 3D model was developed to calculate the inductance *L* and quality factor *Q* of micro-inductor since *L* and *Q* are two important parameters to evaluate an inductor.

### 2.1. Effect of Patterned Magnetic Film on the Performances of Micro-Inductor

**Figure 2:**
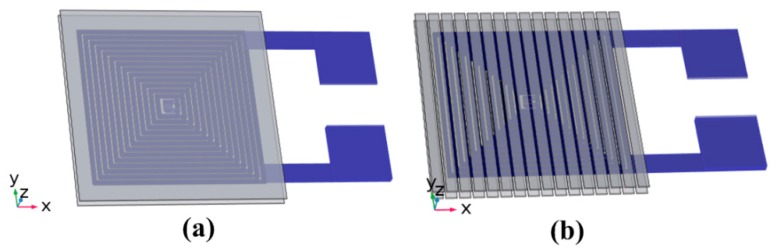
Models of micro-inductors. (**a**) Non-patterned magnetic film; (**b**) patterned magnetic film.

Two 3D models were designed to compare the performances of the inductor, as shown in Figure 2. The simulation was carried out by COMSOL software (COMSOL Multiphysics 5.2a, COMSOL Inc., Stockholm, Sweden). The two models had the same planar coils, magnetic material of Ni_80_Fe_20_ permalloy with a thickness of 10 μm in the same working conditions (the voltage of 5 V and the frequency of 10 k–10 MHz). 

**Figure 3:**
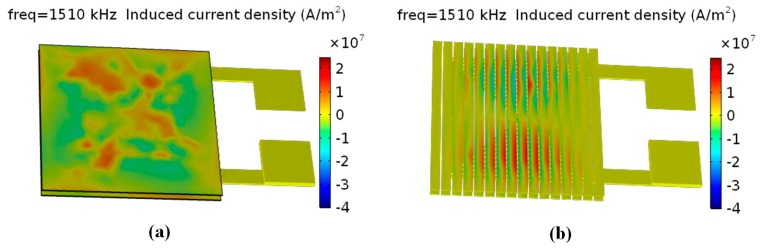
Distribution of surface inductive current density of micro-inductor. (**a**) Non-patterned magnetic film; (**b**) patterned magnetic film.

**Figure 4:**
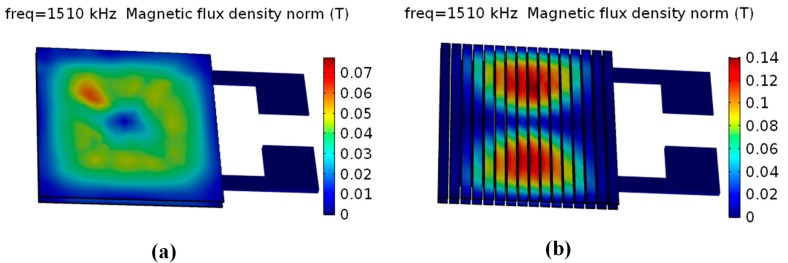
Distribution of surface magnetic flux density of micro-inductor. (**a**) Non-patterned magnetic film; (**b**) patterned magnetic film.

The AC ripple current and the distribution of magnetic flux are closely related to inductance and quality factor. Therefore, the distributions of inductive current density and magnetic flux density at 1.5 MHz for each of the models were calculated. The simulation results were shown in Figure 3 and Figure 4, respectively. As shown in Figure 3, the maximum inductive current density of model (b) (micro-inductor with patterned magnetic film) is obviously reduced when compared with model (a) (non-patterned magnetic film). Model (b) has more low-density-current areas than model (a) as the patterned magnetic film can decentralize the distribution of high current density and increase the area of low current density. That indicates that patterned magnetic film can effectively decrease the eddy current loss of the micro-inductor. In addition, as indicated in Figure 4, both model (a) and model (b) show non-uniform distribution of magnetic flux. However, model (b) shows much higher maximum magnetic flux density than model (a). Hence, it is expected that the micro-inductor with patterned magnetic film can achieve higher inductance and quality factor than that with non-patterned magnetic film.

**Figure 5:**
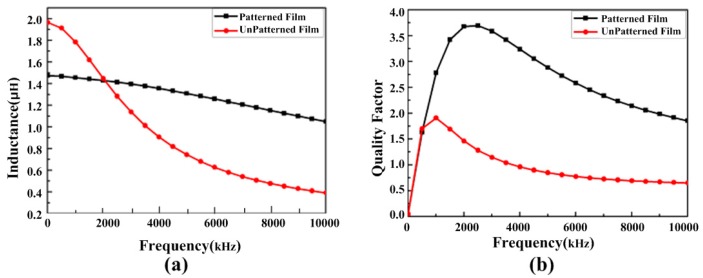
Simulated results for the micro-inductors with patterned or non-patterned magnetic film. (**a**) Inductance as a function of frequency; (**b**) quality factor as a function of frequency.

Figure 5 shows the comparison of the simulated inductance and quality factor of the two models. In Figure 5a, the inductor with patterned magnetic film shows a lower inductance at a low frequency than that with non-patterned magnetic film, because leakage flux results in the reduction of the inductance. Nevertheless, the inductor with non-patterned magnetic film shows more evident eddy current loss than that with patterned magnetic film when the frequency further increases. So the inductor with patterned magnetic film shows higher inductance than that with non-patterned magnetic film at high frequency. In Figure 5b, the micro-inductor with patterned magnetic film has nearly double quality factor in the high frequency range compared to that with non-patterned magnetic film.

Figure 5 shows the comparison of the simulated inductance and quality factor of the two models. In Figure 5a, the inductor with patterned magnetic film shows a lower inductance at a low frequency than that with non-patterned magnetic film, because leakage flux results in the reduction of the inductance. Nevertheless, the inductor with non-patterned magnetic film shows more evident eddy current loss than that with patterned magnetic film when the frequency further increases. So the inductor with patterned magnetic film shows higher inductance than that with non-patterned magnetic film at high frequency. In Figure 5b, the micro-inductor with patterned magnetic film has nearly double quality factor in the high frequency range compared to that with non-patterned magnetic film.

### 2.2. Effect of Magnetic Film Thickness on the Performances of Micro-Inductor

**Figure 6:**
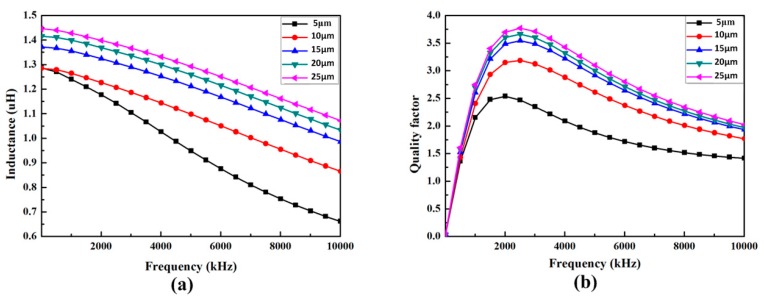
Effect of magnetic film’s thickness on the inductance and quality factor of the micro-inductor. (**a**) Inductance as a function of frequency; (**b**) quality factor as a function of frequency.

The models with different thickness of magnetic film were simulated. The effect of the thickness of magnetic films on the inductance and quality factor of the micro-inductor was investigated. The simulation results are shown in Figure 6a,b, respectively. In Figure 6a, the inductance of the micro-inductor increases with the increasing thickness of the magnetic film. However, the growth rate decreases when the magnetic film is thicker than 15 μm. In Figure 6b, the quality factor of the micro-inductor also increases when the thickness of the magnetic film increases. From the simulation results, it can be concluded that increasing the thickness of the magnetic film is an efficient approach to achieve high inductance and quality factor. 

## 3. Fabrication and Characterization

### 3.1. Fabrication of the Planar Inductor

Three kinds of micro-inductors were fabricated for comparison. The specifications of three micro-inductors were given in Table 1. Type 1 is an inductor without magnetic films, Type 2 is an inductor with non-patterned magnetic film, and Type 3 is an inductor with patterned magnetic film. According to simulated results, the inductance and quality factor increase with the increasing thickness of the magnetic film. In order to achieve high inductance and quality factor, the magnetic film’s thickness of Type 3 is 20 μm.

**Figure 7:**
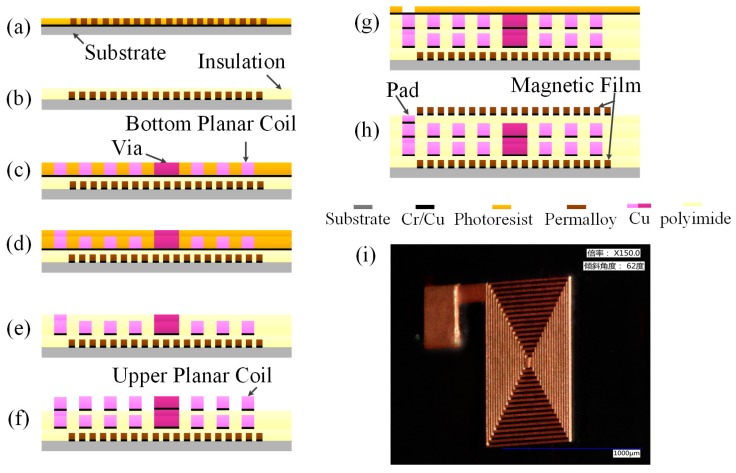
Main fabrication processes of the micro-inductor (**a**)–(**h**) and optical photograph of the single coil (**i**).

Taking Type 3 as an example, the fabrication processes of the micro-inductor are illustrated in Figure 7a–h and described as follows.

(a) The glass wafer was selected as the substrate. Cr/Cu metal was sputtered on the substrate as the first seed layer as the Cr can enhance the adhesive force between Cu and glass substrate. Photoresist was spin coated on the seed layer and patterned by lithography and development. The bottom magnetic film (Ni_80_Fe_20_) was electroplated on Cr/Cu seed layer (thickness 20 µm).
(b) The sodium hydroxide solution (4%) was used to remove the residual photoresist, and an ammonia/peroxide solution was used to remove the chromium/copper seed layers. The polyimide was selected as the insulation layer for filling spacing of the single coil after the photoresist and the seed layer was removed, respectively.
(c) Another Cr/Cu seed layer was sputtered and another layer of photoresist was spin coated and patterned. The bottom planar coil and the via connecting the two layer of coils was fabricated by electroplating process (thickness 20 µm), respectively.
(d) The sodium hydroxide solution (4%) was used to remove the residual photoresist. The third layer of photoresist was spin coated and patterned. The via was further electroplated to a higher position than the bottom coil in order to connect the top coil (Cu 5 µm). The pad was also fabricated by multi-step electroplating process.
(e) The residual photoresist and the Cr/Cu seed layer was removed, respectively. The polyimide was also used as the insulation layer between two layers of the coil.
(f) The third Cr/Cu seed layer was sputtered and the fourth layer of photoresist was spin coated and patterned. The top planar coil was electroplated (thickness 20 µm).
(g) The fourth Cr/Cu seed layer was sputtered and the fifth layer of photoresist was spin coated and patterned to electroplate the pad (Cu 5 µm).
(h) The sixth layer of photoresist was spin coated and patterned. The top magnetic film was electroplated (thickness 20 µm). Acetone was used to remove the positive photoresist, and ammonia/peroxide solution was chosen to remove the seed layer.

Figure 7i shows the optical image of Cu coil with a thickness of 20 µm.

**Figure 8:**
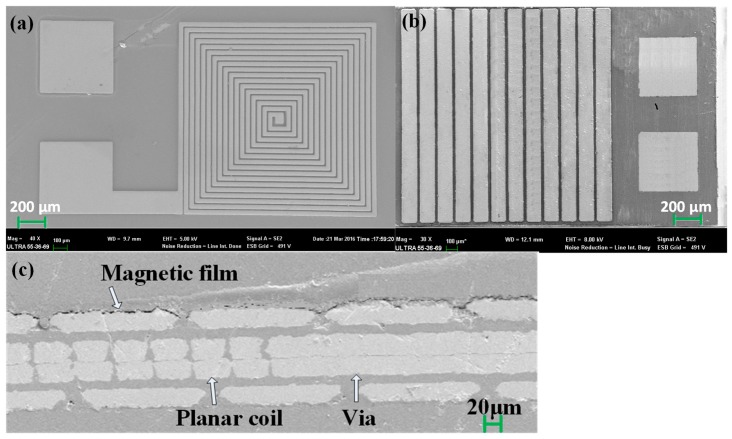
Scanning electron microscope (SEM) images of the fabricated planar inductor with patterned magnetic film. (**a**) Upper Cu coil; (**b**) profile of the mocro-inductor; (**c**) cross section of the micro-inductor.

Figure 8 shows scanning electron microscope (SEM, Carl Zeiss Jena, Jena, Germany) images of the fabricated micro-inductor with patterned permalloy magnetic films. Figure 8a shows SEM image of the upper Cu coils fabricated by electroplating. Figure 8b indicates SEM image of the micro-inductor, where the patterned magnetic film (Ni_80_Fe_20_) was electroplated. Figure 8c shows the cross-sectional images of the fabricated micro-inductor.

### 3.2. Application of the Micro-Inductor in a Buck DC/DC Converter

**Figure 9:**
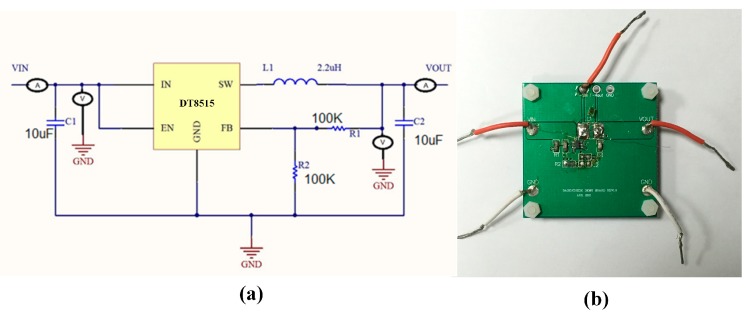
Application circuit of the DC/DC Buck Converter. (**a**) Schematic circuit diagram; (**b**) PCB (Printed Circuit Board) used for testing.

The micro-inductors were developed for its application in a DC/DC buck converter. The DT8515 (Dorsent Technologies Inc., Shanghai, China) was selected as DC/DC converter. The application circuit of the DC/DC buck converter is shown in Figure 9a. A testing Printed Circuit Board (PCB) was designed to enable the high-frequency measurement for the converter and the micro-inductor, as shown in Figure 9b. The fabricated micro-inductors were connected with the PCB by using a wire-bond connection.

## 4. Results and Discussion

### 4.1. Electrical Properties of the Fabricated Micro-Inductors

**Figure 10:**
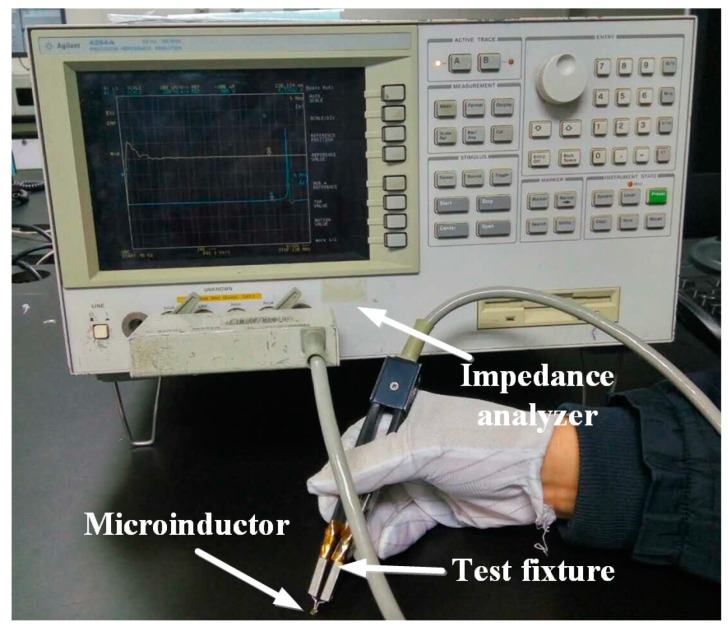
Frequency testing setup for the micro-inductor.

The frequency characteristics of the fabricated micro-inductors were measured by using Agilent E4294A impedance analyzer (Agilent Technologies, Santa Clara, CA, USA) and Agilent16334A test fixture at 0.5 V, as shown in Figure 10.

**Figure 11:**
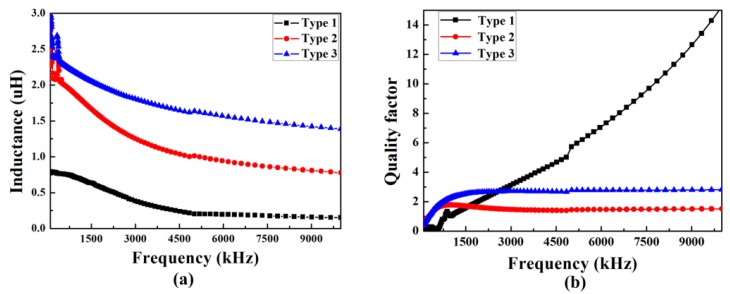
Measured inductances and quality factors of three planar inductors. (**a**) Inductance *L* as a function of frequency; (**b**) quality factor *Q* as a function of frequency.

Figure 11a,b shows the dependence of the inductance *L* and the quality factor *Q* of the three micro-inductors on frequency, respectively. As shown in Figure 11a, although the measured inductance of three micro-inductors decreased gradually with the increase of frequency, the inductance of Type 3 with patterned magnetic films is 2.17 μH at 1.5 MHz, which is three times larger than that of Type 1. The maximum quality factor of Type 3 is about 2.8 at 1.5 MHz. The inductance of Type 3 is much larger than that of Type 2 because eddy current loss is reduced for the patterned magnetic films. In Figure 11b, the micro-inductor with patterned magnetic film is nearly two times the quality factor of that with non-patterned magnetic film.

### 4.2. Characteristics of Analog Circuit Testing 

**Figure 12:**
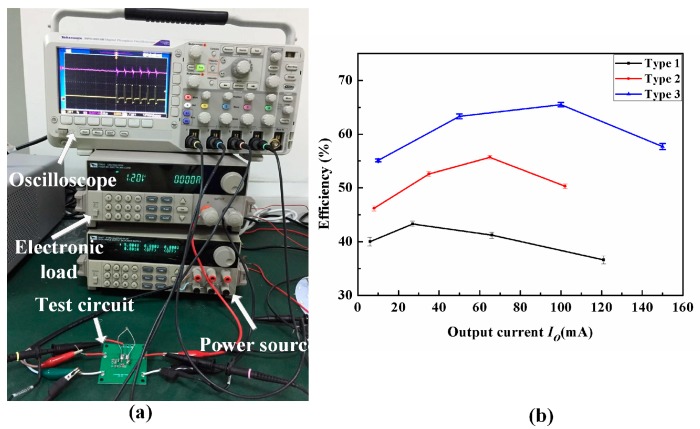
(**a**) Testing setup; (**b**) Efficiency versus output current of the DC/DC buck converter using fabricated micro-inductors.

The fabricated inductors were tested by an electronic system, as shown in Figure 12a. The ITECH DC ELECTRONIC LOAD (IT8510, 120 V/20 A/120 W, ITECH, Yorba Linda, CA, USA) was used as the simulation load. Agilent 6000 oscilloscope (MSO6034A, Agilent Technologies, Santa Clara, CA, USA) was used to collect the output voltage. ITECH TRIPLE OUTPUT DC POWER SUPPLY (IT6322, 0–3 V) was used for the power supply providing typical converter parameters, such as 5 V input voltage, 0–1.2 V output voltage, 100 mA load DC current. The measured efficiency versus output current of the 1.5-MHz switching buck DC/DC converter (*Vi* = 5 V, *Vo* = 1.2 V) is shown in Figure 12b. For Type 3 micro-inductor, the converter has a 67% maximum efficiency at an output current of 100 mA. The energy loss of Type 3 decreases by 15% compared to that of Type 2.

As shown in Figure 11 and Figure 12, the micro-inductor with patterned magnetic film has the improved inductance and quality factor compared to that without magnetic film or with non-patterned magnetic film. The patterning of magnetic film is useful in controlling the distribution of magnetic field and current according to the simulation results, which is closely related to the inductance and quality factor of the inductor. The measured results are consistent with the simulation results. 

## 5. Conclusions

The micro-inductor has been successfully developed by using patterned permalloy thin-film. The effects of magnetic film and its thickness on the performances of the micro-inductor were simulated. Simulation results show that the patterning of the magnetic film effectively improves the inductance and quality factor of the micro-inductor. The inductance and quality factor of the fabricated inductors in high frequency range (>1 MHz) were measured by Agilent E4294A. The inductance of Type 3 is approximately four times greater than Type 1, and the quality factor of Type 3 at 1.5 MHz is enhanced by 64% compared to that of Type 2. The converter with the fabricated micro-inductor has a 67% maximum efficiency at an output current of 100 mA, and has a margin of error of plus or minus 0.6 percent. The micro-inductor with a height of 100 μm is beneficial to packaging requirements for DC/DC integration.

## Figures and Tables

**Table 1:** Specifications of three planar inductors.

Types	Copper Square Spiral Coil				Magnetic Films		
	Number of Tums (*N*)	Thickness (*t_c_*)	Line Width (*w_c_*)	Spacing (*S*)	Material (*M*)	Thickness (*t_s_*)	Outer Dimension
**1**	17	20 μm	20 μm	20 μm	-	-	-
**2**					Permalloy	10 μm	1.9 mm × 1.9 mm
**3**					Permalloy	20 μm	1.9 mm × 0.12 mm × 12 mm
